# Comparison of Selected Phenotypic Features of Persistent and Sporadic Strains of *Listeria monocytogenes* Sampled from Fish Processing Plants

**DOI:** 10.3390/foods11101492

**Published:** 2022-05-20

**Authors:** Natalia Wiktorczyk-Kapischke, Ewa Wałecka-Zacharska, Krzysztof Skowron, Agnieszka Kijewska, Zuzanna Bernaciak, Justyna Bauza-Kaszewska, Zuzanna Kraszewska, Eugenia Gospodarek-Komkowska

**Affiliations:** 1 Department of Microbiology, L. Rydygier Collegium Medicum in Bydgoszcz, Nicolaus Copernicus University in Toruń, 85-094 Bydgoszcz, Poland; natalia12127@gmail.com (N.W.-K.); skowron238@wp.pl (K.S.); zuza.benciak@gmail.com (Z.B.); zuzia95@op.pl (Z.K.); gospodareke@cm.umk.pl (E.G.-K.); 2 Department of Food Hygiene and Consumer Health, Wrocław University of Environmental and Life Sciences, 50-375 Wrocław, Poland; 3 Department of Immunobiology and Environmental Biology, Institute of Maritime and Tropical Medicine, Medical University of Gdansk, 80-210 Gdańsk, Poland; agnieszka.p.kijewska@gumed.edu.pl; 4 Department of Microbiology and Food Technology, Jan and Jędrzej Śniadecki University of Technology in Bydgoszcz, 85-029 Bydgoszcz, Poland; justynabauza@gazeta.pl

**Keywords:** *Listeria monocytogenes*, persistent strains, sporadic strains, fish processing, resistance to disinfectants, biofilm, foodborne microorganism

## Abstract

(1) Background: The main source of transmission of *Listeria monocytogenes* is contaminated food, e.g., fish and meat products and raw fruit and vegetables. The bacteria can remain for 13 years on machines in food processing plants, including fish plants. (2) Methods: A total of 720 swabs were collected from a salmon filleting line. The research material consisted of 62 (8.6%) *L. monocytogenes* isolates. Pulsed Field Gel Electrophoresis (PFGE) allowed detecting a pool of persistent strains. All persistent strains (*n* = 6) and a parallel group of strains collected sporadically (*n* = 6) were characterized by their ability to invade HT-29 cells, biofilm formation ability, and minimum bactericidal concentrations (MBC) of selected disinfectants. (3) Results: Among the obtained isolates, 38 genetically different strains were found, including 6 (15.8%) persistent strains. The serogroup 1/2a-3a represented 28 strains (73.7%), including the persistent ones. There were no significant differences in invasiveness between the persistent and sporadic strains. The persistent strains tolerated higher concentrations of the tested disinfectants, except for iodine-based compounds. The persistent strains initiated the biofilm formation process faster and formed it more intensively. (4) Conclusions: The presence of persistent strains in the food processing environment is a great challenge for producers to ensure consumer safety. This study attempts to elucidate the phenotypic characteristics of persistent *L. monocytogenes* strains.

## 1. Introduction

*Listeria monocytogenes* is a rod-shaped, Gram-positive, and widespread in the environment bacterium. *L. monocytogenes* adapts to unfavorable conditions in food processing plants. This pathogen is vulnerable to nutrient deficiency, heat shock, high osmolarity, and low pH [1,2,3,4]. The most common sources of *L. monocytogenes* are ready-to-eat (RTE) food and fish products, meat, poultry, raw milk, soft raw milk cheese, fresh and frozen vegetables, and packed salads. Fish is a common source of *L. monocytogenes*. The contamination of finished products may occur during the production process, during such activities as filleting, rinsing, and salting [2,5,6,7,8,9]. *L. monocytogenes* is the etiologic factor of a severe food-borne disease—listeriosis. Pregnant women, immunocompromised individuals, and the elderly are very sensitive groups to *Listeria* infections [1,10]. Despite its low incidence, the high fatality rate (15–20%) makes listeriosis a serious food-borne disease [11]. A recent listeriosis outbreak, with 200 deaths, was noted in Africa (2017–2018) and was due to RTE product consumption [12]. The presence of *L. monocytogenes* in food may be the effect of the contamination of raw materials or processed products at different stages of the production chain [13,14]. Point 22 of EU Regulation 2073/2005 aims at food safety assurance by testing samples from the food environment [15]. One serious problem is the emergence of persistent strains, despite the undertaken hygiene interventions. There is no unified, clear definition of the persistent strains and they are determined subjectively for individual research. So, if the subset of the bacterial population can survive exposure to a higher than usually used bactericidal drug concentration, it may be referred to as persistence, according to the definition given by Balaban et al. [16]. Presumably, the same strain recurrently detected over a specific period confirms the emergence of persistent strains [1]. The extreme period was noted by Fagerlund et al. [17] and lasted for 13 years. Stable strains of *L. monocytogenes* are indistinguishable pulsotypes (bacteria separated by pulsed field gel electrophoresis, PFGE), but their characteristics, both in terms of PFGE metric and serogroup, remain the same over time and maintain their properties, including a resistance to certain biocides [18]. In different studies, the persistence time varied from months to 12 years [3,19,20] and bacteria remained to be dangerous for potential consumers. For instance, a *L. monocytogenes* strain considered persistent was sampled from an Estonian company’s premises, which produced cold smoked salmon and trout sold in countries of the European Union. The outbreak caused by the presence of this persistent strain of *L. monocytogenes* (sequence type (ST) 1247) in food included 22 cases of listeriosis in five EU countries [21].

Several concepts have been suggested to describe the strains’ persistence. Resistance to stress factors is the most important, followed by biofilm formation and increased tolerance to disinfectants [4]. The most important factor influencing the persistence of bacteria is the high resistance to stress factors, such as pH, temperature, specificity and limitation of nutrient sources, and competition with other microorganisms. The pH of the fish is usually alkaline, and salt is also used as a preservative in the food industry [22,23,24]. The capability to grow at a low temperature and high salt concentrations promotes *L. monocytogenes* survival in the production environment.

The relevant source of the pathogen in the food processing environment is the reintroduction of persistent strains from external habitats [3]. *L. monocytogenes* has many adaptive mechanisms enabling its survival in adverse environmental conditions, including food processing [25]. Some studies suggest a higher adherence of persistent bacteria to food contact surfaces than the nonpersistent strains [26].

Researchers have documented higher biofilm formation ability among persistent, compared to nonpersistent, strains [27]. Biofilms are considered a source of persistent pathogenic microorganisms [10]. Moreover, the increased persistence of pathogenic bacteria can be the effect of co-existence with other, non-pathogenic microorganisms in multispecies biofilms [3]. *L. monocytogenes* in the biofilm revealed increased resistance to disinfecting agents compared to planktonic form. Adaptation and resistance to disinfectants, developed after *L. monocytogenes* exposition to their sublethal concentrations, can also affect the prolonged survival of the bacteria in the food processing environment [8,24,28].

The research aims to evaluate the differences in selected phenotypic properties between the persistent and sporadic strains of *L. monocytogenes* collected along the entirety of the fish processing line.

## 2. Materials and Methods

### 2.1. Sampling Procedure

From April to September 2019, 720 swabs (120 per month) were collected from machines and surfaces used for fish fillet production at one plant located in East-Central Europe, the north Poland. Samples were collected between work shifts, after cleaning procedures, when machines were not working.

To collect bacteria, the wet swab method was used. A sterile, flexible template limiting the tested area to 100 cm^2^ was used. Samples were taken from the box pallets in the raw material warehouse (60 swabs), fish head remover machine (60 swabs), filleting machine (conveyor—60 swabs; knives—60 swabs), trim conveyor (conveyor rollers—60 swabs; worktops—60 swabs), pin bone remover (conveyor—60 swabs; trommel—60 swabs), fish skin remover machine (60 swabs), fillet washer (60 swabs) and portion machine (conveyor—60 swabs; knives—60 swabs). A sterile 50 cm² cellulose sponge (Enviroscreen, Technical Service Consultants Ltd., Lancashire, UK) soaked in 10 mL of a sterile 0.9% NaCl packed in a reinforced zip-bag was used for sampling.

### 2.2. Sampling and Identification of L. monocytogenes Isolates

The analysis of the samples was based on ISO 11290-1 procedures [29]. The swabs taken from the surface of machines were immersed in 100 mL of half-Fraser broth (Merck, Darmstadt, Germany) and incubated at 30 °C for 24 h. Secondary selective enrichment was performed for 48 h at 37 °C after transferring 0.1 mL of the culture into 9.9 mL of Fraser broth (Merck). Next (both after incubation in half-Fraser broth and Fraser broth), bacteria were plated on the selective agar medium according to Ottaviani and Agosti (ChromoCult^®^ Listeria Selective Agar, ALOA^®^, Merck) and incubated for 24 h at 37 °C. Selected colonies, initially identified according to the manufacturer’s recommendations as *Listeria* spp., were transferred to Columbia Agar with 5% sheep blood (bioMérieux, Marcy-l’Étoile, France).

Finally, the MALDI-TOF MS (Matrix-Assisted Laser Desorption and Ionization—Time of Flight Mass Spectrometry) technique was used to confirm if presumptive colonies belonged to the *L. monocytogenes* species. The acquisition and analysis of mass spectra were performed by a Microflex LT/SH mass spectrometer (Bruker, Billerica, MA, USA) using the MALDI Biotyper software package (version 4.1) with the Bruker Taxonomy reference database (Bruker). The ethanol–formic acid extraction procedure was applied for samples preparation. The bacterial test standard (BTS; Bruker) was used for validation according to the manufacturer’s instructions.

The identified *L. monocytogenes* isolates were frozen in a brain–heart infusion broth (BHI, Merck) with 15% glycerol (Avantor, Gliwice, Poland) and stored at −80 °C.

### 2.3. Assessment of the Genetic Similarity of the Collected Isolates

The genetic similarity analysis of the confirmed *L. monocytogenes* strains was performed with the pulsed-field gel electrophoresis (PFGE), which is the golden standard to identify putative routes of contamination and persistent strains according to Dalmasso and Jordan [30]. The procedure for genotyping was performed following the Standard Operating Procedure for PulseNet PFGE of *Listeria monocytogenes* (PNL04, last updated April 2014) [31]. The ApaI enzyme was used in the study. The electrophoretic separation was performed with the following parameters: initial and final pulse duration: 4–40 s; voltage: 6 V/cm; pulse angle: 120°; temperature 14 °C; program duration: 17 h. The degree of genetic similarity between the analyzed *L. monocytogenes* isolates was evaluated using a phylogenetic dendrogram drawn in the CLIQS 1D Pro program (TotalLab, Newcastle upon Tyne, UK). Clustering analysis was performed using hierarchical clustering with the UPGMA technique and Dice’s coefficient. The cut-off value to define the PFGE patterns was set at 80% similarity. The isolates were considered as genetically identical when identical pulsotypes were demonstrated for them by the PFGE method.

### 2.4. Isolation of Genomic DNA

Isolation of genomic DNA was performed using the Genomic Mini AX Bacteria Spin Kit (A&A Biotechnology, Gdańsk, Poland), according to the manufacturer’s procedure, and the DNA was stored at −20 °C for further analyses.

### 2.5. Determination of Serological Groups

Multiplex PCR for the identification of the main *L. monocytogenes* serogroups (1/2a-3a, 1/2b-3b, 1/2c-3c, 4b-4d-4e) was performed as described by Doumith et al. [32]. The PCR was performed on a cycler Mastercycler^®^ pro (Eppendorf, Hamburg, Germany) using: 1.5 ×  PCR buffer (Promega, Madison, WI, USA), 2 mM MgCl_2_ (Promega), 1.25 mM dNTPs (Promega), 0.5 μM of each primer (Oligo.pl, Warszawa, Poland), 1 U GoTaq DNA polymerase (Promega), ultrapure water (Sigma Aldrich, Saint Louis, MO, USA), and the previously isolated genomic DNA. The amplicons were electrophoretically separated in 1.5% agarose gel (Sigma Aldrich) stained with Midori Green (NIPPON Genetics EUROPE GmbH, Düren, Germany) in 1 × TBE buffer (BioRad, Hercules, CA, USA), in the presence of a DNA size standard (GeneRulerTM1000 bp DNA Ladder) (Fermentas, Waltham, MA, USA) (90 V, 1 h).

For each PCR reaction, the four selected *L. monocytogenes* strains examined by Wałecka-Zacharska et al. [33] were used as positive control strains for serogroup identification. The negative control in each reaction was a sample without DNA.

### 2.6. Assessment of Drug Susceptibility of the Tested L. monocytogenes Strains

The selected antibiotics were among those frequently used in the first-line treatment of *L. monocytogenes* infections in humans and those also used in the veterinary treatment of farm animals. This is important as Poland is one of the largest producers and exporters of meat and dairy products in the European Union [34]. The evaluation of drug susceptibility was performed for genetically different isolates (62) using the disk diffusion method on the Mueller–Hinton agar with 5% defibrinated Horse Blood and 20 mg/L β-NAD (MH-F, bioMérieux). Disks with penicillin (1 IU), ampicillin (2 µg), meropenem (10 µg), erythromycin (15 µg), and cotrimoxazole (1.25–23.75 µg) were used. Antibiograms were incubated in an atmosphere enriched in 5% CO_2_ at 35 °C for 18 h. The results were interpreted, according to the recommendations of EUCAST (European Committee on Antimicrobial Susceptibility Testing) v. 12.0. [35].

### 2.7. Comparison of the Selected Properties of Sporadic and Persistent L. monocytogenes Strains

At this stage of the study, 6 persistent strains (LMO-P1, LMO-P2, LMO-P3, LMO-P4, LMO-P5, and LMO-P6) and 6 sporadic strains (LMO 4, LMO 23, LMO 46, LMO 52, LMO 53, and LMO 61) were selected. The following tests were repeated in triplicate for each isolate. Each repetition consisted in the independent preparation of a new bacterial suspension for a given strain and the performance of all tests described in the methodology.

#### 2.7.1. Assessment of Invasiveness against HT-29 Eukaryotic Cells

Tested strains were plated on Columbia Agar with 5% sheep blood (bioMérieux) and incubated for 24 h at 37 °C. Single colonies were transferred into 5 mL brain–heart infusion broth (BHI, Merck) and incubated in a thermoblock (TDB-100, Biosan, Józefów, Poland) at 37 °C (230 rpm, 6 h). Next, 5 µL of the bacterial suspension was transferred into 5 mL of BHI broth and incubated another 18 h until an OD600 of 2.4–2.6 was obtained (measured with the DU 8800D spectrophotometer). The bacteria of 5–6 log CFU (Colony Forming Units) were used to infect the human colon carcinoma HT-29 cell line (CLS, Germany).

HT-29 cells were seeded in 6-well polystyrene culture plates (Genoplast) and incubated to approx. 90% confluence in Dulbecco’s Modified Eagle Medium (DMEM, Sigma-Aldrich), containing 10% fetal bovine serum (FBS, Gibco, Park Ridge Ln S Billings, MT, USA), 2 mM glutamine, and 100 IU/mL penicillin and 100 µg/mL streptomycin (Sigma-Aldrich). Before the cells’ infection (24 h), the medium was changed to DMEM without antibiotics. The HT-29 cells were incubated with bacteria for 2 h (37 °C, 5% CO_2_). The wells were then washed twice with a sterile PBS solution (Sigma-Aldrich) and incubated in DMEM containing 100 µg/mL gentamycin (Sigma-Aldrich) for 1.5 h (37 °C, 5% CO_2_). Next, the wells were washed twice with PBS and overlaid with a medium containing 10 µg/mL gentamicin and 1.0% low melting point agarose (Prona, Gdańsk, Poland). After 48 h of incubation, the number of plaques was determined. Bacterial invasiveness was calculated as the quotient of the number of plaques (expressing the number of bacteria that entered HT-29 cells) and the number of bacteria introduced into the wells. Invasiveness was expressed as a percentage.

#### 2.7.2. Determination of the Minimum Bactericidal Concentration (MBC) of Selected Disinfectants against Persistent and Sporadic Strains of *L. monocytogenes*

The evaluation of the minimum bactericidal concentrations (MBC) for the selected disinfectants was previously described by Skowron et al. [36]. Table 1 presents the disinfectants included in the study.

The bacterial suspensions (100 μL) and 100 μL of the appropriate concentration of disinfectant were added to the 96-well polystyrene plate. The final concentrations of the disinfectants were: 100%, 90%, 80%, 70%, 60%, 50%, 40%, 30%, 20%, 10%, 5%, 1%, and 0.5% of the working solution. The negative control consisted of 200 μL of a sterile MHB (Mueller–Hinton Broth, Becton Dickinson) medium, and the positive control 200 μL of the bacterial suspension. After 5 min of the agent’s action, 100 μL of each suspension was transferred into 900 μL of neutralizer (10 g Tween 80 (Sigma Aldrich), 1 g lecithin (Sigma Aldrich), 0.5 g histidine L (Sigma Aldrich), 2.5 g Na_2_S_2_O_3_ (Avantor), 3.5 g C_3_H_3_NaO_3_ (Avantor), and 1000 mL sterile water)). After 5 min of neutralization, samples were inoculated onto Columbia Agar with 5% sheep blood (bioMérieux). After 24 h incubation at 37 °C, the bacterial growth and the MBC value were assessed.

After determining the MBC range, the procedure was repeated with solutions at concentrations varying by 1% from the designated MBC value. This procedure allowed for the exact determination of the MBC value.

#### 2.7.3. Assessment of the Rate of Initiation of Biofilm Formation

Biofilm formation ability was assessed on stainless steel coupons (1 cm × 2 cm, AISI 304 type). Coupons were washed in a commercial detergent, soaked for 5 min in 70% ethanol (Avantor), and autoclaved. For each strain, 5 coupons for one experiment were prepared. The research was carried out in triplicate.

Sterile steel coupons were placed in tubes containing 3 mL of bacterial suspension (0.5 McF) in BHI (Merck) and incubated in aerobic atmosphere at 37 °C for 1, 2, 3, 4 and 5 h, respectively. After incubation, the samples were rinsed with PBS solution and placed in a tube containing 3 cm^3^ of this solution. Next, sonication (10 min, 30 kHz, 150 W) was performed using the Ultrasonic DU-4 (Nickel-Electro Ltd., Oldmixon Cres, Weston-super-Mare BS24 9BL, UK) sonicator.

After sonication, serial 10-fold dilutions of the obtained suspension in sterile PBS were prepared, plated on the Columbia Agar medium with 5% Sheep Blood (Becton Dickinson, Franklin Lakes, NJ, USA), and incubated for 24 h at 37 °C. For the group of persistent and sporadic strains, the mean number of *L. monocytogenes* recovered from the coupon surface after a given incubation time was calculated. The results were presented as the log CFU × cm^−2^.

#### 2.7.4. Assessment of the Intensity of Biofilm Formation

Sterile steel coupons were placed in tubes containing 3 mL of suspension of each strain (0.5 McF) in BHI of selected parameters (pH, salinity, and availability of nutrients), and were incubated for 72 h (Table 2). During incubation for each strain, except for experimental condition 1 (low temperature growth), the medium was replaced every 24 h with a fresh one and the coupons were rinsed with sterile PBS. For the variant 1 (4 °C) strain, the medium was replaced every 4 days and the incubation was extended to 12 days. As a negative control, steel coupons in a sterile BHI, in variables set up appropriately, were used. The first CFU’s counting was performed after 1 day of incubation (in the case of low temperature, after the 4th day of incubation).

After incubation, the samples were rinsed with a PBS solution and placed in a tube containing 3 mL of this solution. Next, sonication (10 min, 30 kHz, 150 W) was performed using the Ultrasonic DU-4 (Nickel-Electro Ltd.) sonicator.

After sonication, serial 10-fold dilutions of the obtained suspension in sterile PBS were prepared, plated on Columbia Agar medium with 5% Sheep Blood (Becton Dickinson), and incubated for 24 h at 37 °C. For the group of persistent and sporadic strains, the mean number of *L. monocytogenes* recovered from the coupon surface under given environmental conditions was calculated. The results are presented as the log CFU × cm^−2^.

### 2.8. Statistical Analysis

The statistical analysis was carried out in the STATISTICA 13.0 PL (TIBCO Software, Palo Alto, CA, USA) software. With the use of general linear models (GLM) and ANOVA analysis, the statistical significance of differences was checked at the level of α = 0.05. Based on one-way ANOVA, the differences in the percentages of all strains tested representing a given serogroup, resistance to a given antibiotic, and a given drug’s susceptibility profile were checked. The significance of differences in invasiveness between the persistent and nonpersistent strains, MBC values for each of the tested disinfectants, and the intensity of biofilm formation over time were also checked. In turn, based on the multivariate ANOVA, the differences in the number of bacteria recovered from the biofilm between the persistent and nonpersistent strains, depending on the conditions of its formation, were checked.

## 3. Results

**Figure 1:**
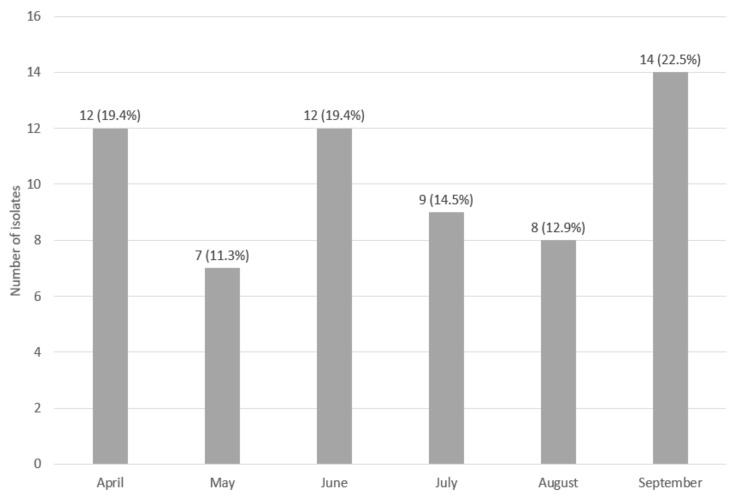
Number and percentage (%) of the total number of obtained isolates (62–100%) of *L. monocytogenes* according to months of sampling.

Out of 720 swabs taken from the surface of the fish processing machines, 62 isolates were identified as *L monocytogenes* (8.6%) (Table 3). We collected the highest number of isolates (14, 22.6%) from the filleting machine knives and the least (3, 4.8%) from box pallets in the raw material warehouse, the filleting machine conveyor, the worktops of the trim conveyor, the pin bone remover conveyor, the fillet washer, and the portion machine knives (Table 3). We obtained the highest number of isolates (14, 22.6%) in September and the lowest number (7, 11.3%) in May (Figure 1).

### 3.1. Assessment of the Genetic Similarity of Isolate Strains Classified as Persistent

The assessment of the genetic similarity of the isolates allowed the selection of the persistent strains. Persistent strains were defined as strains represented by genetically identical isolates obtained from a particular part of the processing line at least three times over six months.

**Figure 2:**
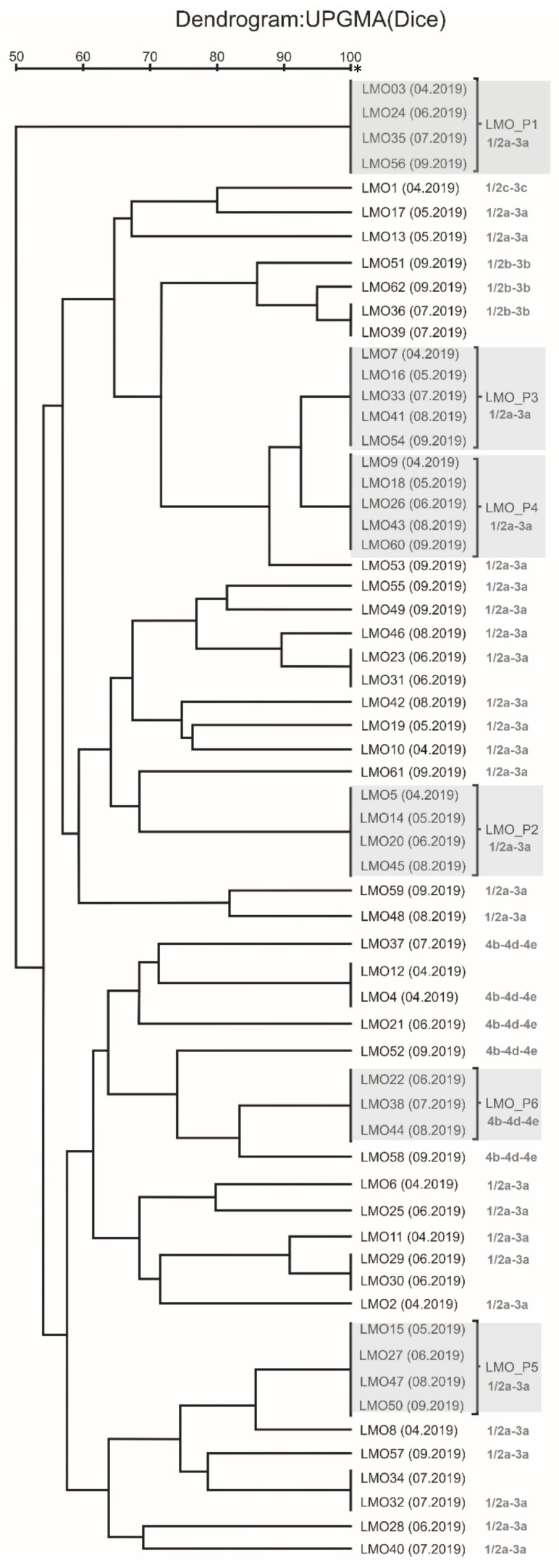
Genetic similarity dendrogram of each tested isolate with clusters of isolates belonging to each persistent strain (marked by gray blocks). * isolates genetically indistinguishable.

Among the 62 obtained isolates, 38 genetically different strains of *L. monocytogenes* were found (Figure 2). The cut-off level equal to 80% allowed to identify 14 strains with a different number of isolates. Twelve isolates corresponded to single-member clusters. The existence of 5 pairs of genetically identical isolates collected at the same time of sampling was demonstrated (Figure 2).

Six persistent strains meet the criterion adopted in the research methodology (Figure 2). The genetically identical isolates obtained at different times, but belonging to a given persistent strain, are listed in brackets:

- LMO-P1 (LMO 3 (April 2019), LMO 24 (June 2019), LMO 35 (July 2019), and LMO 56 (September 2019))—isolated from the trim conveyor rollers.
- LMO-P2 (LMO 5 (April 2019), LMO 14 (May 2019), LMO 20 (June 2019), and LMO 45 (August 2019))—isolated from the cutting element of the head remover machine.
- LMO-P3 (LMO 7 (April 2019), LMO 16 (May 2019), LMO 33 (July2019), LMO 41 (August 2019), and LMO 54 (September 2019))—isolated from the knives of the filleting machine.
- LMO-P4 (LMO 9 (April 2019), LMO 18 (May 2019), LMO 26 (June 2019), LMO 43 (August 2019), and LMO 60 (September 2019))—isolated from the knives of the filleting machine.
- LMO-P5 (LMO 15 (May 2019), LMO 27 (June 2019), LMO 47 (August 2019), and LMO 50 (September 2019))—isolated from the skin remover machine.
- LMO-P6 (LMO 22 (June 2019), LMO 38 (July 2019), and LMO 44 (August 2019))—isolated from the trommel of the pin bone remover.

Each isolate constituting a given persistent strain was obtained from the same place in the processing line, but at different sampling times.

In this pool of strains, the LMO-P3 and LMO-P4 strains were genetically similar at 93%, while the LMO-P1 strain was the most genetically distant (Figure 2).

### 3.2. Molecular Serotyping of L. monocytogenes Strains

The analysis of DNA patterns on agarose gel showed 5 persistent strains belonging to the 1/2a-3a serogroup represented by 28 (73.7%) of all detected strains, while the 6th persistent strain (LMO-P6) belonged to 4b-4d-4e serogroup. In turn, only one strain, LMO-1 representing 2.6% of all strains, belonged to the 1/2c-3c serogroup (Figure 2).

### 3.3. Assessment of Drug Susceptibility of the Tested L. monocytogenes Strains

**Figure 3:**
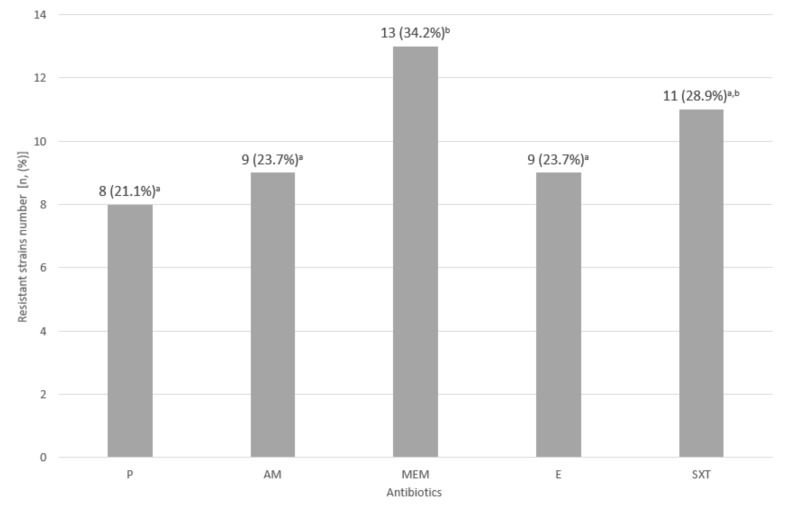
Resistance of 38 isolates of *L. monocytogenes* to antibiotics (P—penicillin; AM—ampicillin; MEM—meropenem; E—erythromycin; SXT—cotrimoxazole; ^a^,^b^—values marked with different letters differ in a statistically significant way (*p* ≤ 0.05). In brackets, the percentage of resistant isolates is given. The limited number of isolates is a result of the exclusion of genetically identical isolates.

Among the *L. monocytogenes* isolates resistant to at least one antibiotic, the greatest number of isolates (13, 34.2%) were resistant to meropenem. In turn, resistance to penicillin was the least common (8, 21.1%) (Figure 3).

The conducted experiment allowed for the identification of six antibiotic resistance profiles. The isolates representing profile no. 1 (18 isolates, 47.4%) (Table 4) were susceptible to all tested antibiotics. The remaining 20 (52.6%) individual isolates and those representing persistent strains showed resistance to at least one tested antibiotic. Counting isolates without persistent strains, 39 of all isolates were resistant to at least one antibiotic. Persistent strains belonged to profiles no. 3 (LMO-P1 and LMO-P2—resistant to meropenem, erythromycin, and cotrimoxazole), no. 4 (LMO-P6—resistant to penicillin and meropenem), and no. 5 (LMO-P3, LMO-P4, and LMO-P5—resistant to all tested antibiotics) (Table 4). Part of single isolates represented an identical resistance to antibiotics as persistent strains.

### 3.4. Assessment of Invasiveness against HT-29 Eukaryotic Cells

The invasiveness of the persistent *L. monocytogenes* strains ranged from 1.07% for LMO-P6 to 11.21% for LMO-P4. In turn, the invasiveness of sporadic strains ranged from 1.42% for LMO 4 to 7.99% for LMO 61. The mean invasiveness calculated for the persistent strains was slightly higher than for the sporadic strains (5.50% vs. 4.60%); however, the difference was not statistically significant (Table 5).

### 3.5. Minimum Bactericidal Concentrations (MBC) of the Selected Disinfectants against the Persistent and Sporadic Strains of L. monocytogenes

For all strains, both persistent and sporadic, the effective concentrations of disinfectants were lower than the concentrations of the working solution recommended by the manufacturer (Table 6). MBC values depended on the properties of a particular strain and the type of disinfectant.

For each strain belonging to the persistent strain group, the MBC values were higher than for strains from the sporadic strain group. For the persistent strains, the values ranged from 33% (0.99 mL/L) of the working solution concentration for Sansept 0200 to 74% (3.71 mL/L) of the working solution concentration for Peroxat (Table 6). In turn, for sporadic strains, the MBC values ranged from 7% (0.22 mL/L) of the working solution concentration for Sansept 0200 to 44% (2.19 mL/L) of the working solution concentration for Peroxat (Table 6). For each tested disinfectant, except Rapicid, statistically significant differences were found between the MBC values established for persistent and sporadic strains (Table 6). In the pool of persistent strains, the LMO-P4 strain turned out to be the most resistant, and the most susceptible one was LMO-P6 (Table 6). In turn, among sporadic strains, the most resistant was LMO 61 and the most sensitive was LMO 4 (Table 6).

Sansept 0200 turned out to be the most effective disinfectant for strains from both groups, and Peroxat the least effective (Table 6).

### 3.6. Assessment of the Rate of Initiation of Biofilm Formation

**Figure 4:**
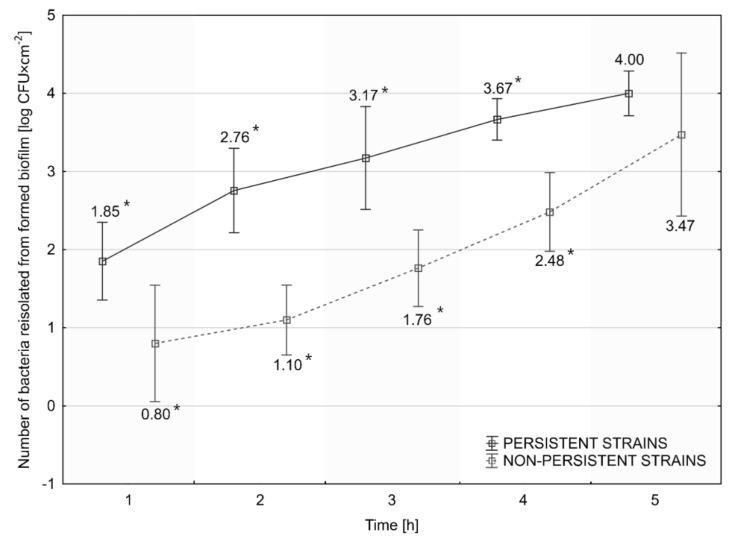
Assessment of the rate of initiation of biofilm formation for the persistent and nonpersistent *L. monocytogenes* strains. The horizontal shift of points relative to the timeline is only used to maintain the chart’s readability. The starting density at the 0 h timepoint was 0.5 McF for each strain. *—values assigned to the same time point, marked with an asterisk, differ statistically significantly (*p* ≤ 0.05).

Persistent *L. monocytogenes* strains formed biofilm faster than sporadic strains (Figure 4). The number of *L. monocytogenes* recovered from the biofilm after 1, 2, 3, and 4 h was statistically significantly higher for persistent than for sporadic strains. No statistically significant differences were found at the 5th h of biofilm formation, although the number of rods recovered remained higher for persistent strains (Figure 4). We noticed the largest difference in the number of recovered *L. monocytogenes* between persistent and sporadic strains, amounting to 1.66 log CFU at the 2nd h of biofilm formation (Figure 4).

### 3.7. Assessment of the Intensity of Biofilm Formation

**Figure 5:**
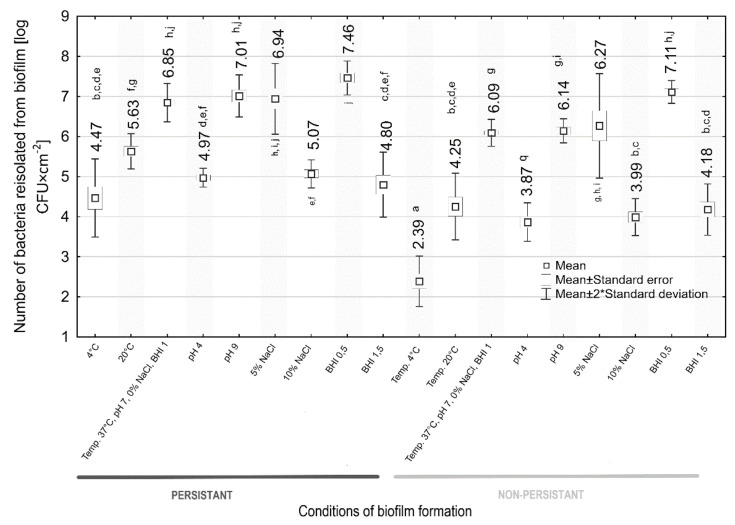
Assessment of the intensity of biofilm formation for the persistent and nonpersistent *L. monocytogenes* strains according to single variable changes being given along X-ordinate. Control parameters were 37 °C, pH 7, 0% NaCl, 1 BHI. a–j: values marked with different letters differ statistically (*p* ≤ 0.05).

The effect of environmental conditions on biofilm formation by both persistent and sporadic *L. monocytogenes* strains was shown (Figure 5). We observed statistically significant differences in the number of *L. monocytogenes* recovered from biofilm between the persistent and sporadic strains (6.85 vs. 6.09 log CFU × cm^−2^) under the control conditions (37 °C, pH 7, 0% NaCl, 1 BHI) (Figure 5).

Analyzing the effect of temperature, we found that the strains from both groups showed a weaker biofilm-forming capacity at 4 °C and performed best at 37 °C. The number of bacteria recovered from the biofilm under all temperature conditions was statistically significantly higher for the persistent strains (Figure 5).

Both persistent and sporadic strains formed a weak biofilm at pH 4 and a one stronger at pH 9 compared to the biofilm formation in the control conditions (Figure 5). At the tested pH values, the number of *L. monocytogenes* obtained from biofilms was statistically significantly higher in the case of persistent strains than sporadic ones (Figure 5).

All strains formed biofilms less intensely in increased salinity (5% and 10% NaCl) compared to what was formed in the control conditions (Figure 5). The bacteria performed better at creating biofilm at 5% NaCl than 10% NaCl (Figure 5). Regardless of salinity, the number of *L. monocytogenes* recovered from biofilm was higher for persistent strains, with a statistically significant difference only shown for 10% NaCl salinity (Figure 5).

The reduced availability of nutrients (0.5 BHI) increased the intensity of biofilm formation in both strain groups (Figure 5). In turn, the increased availability of nutrients (1.5 BHI) lowered the biofilm formation intensity (Figure 5). The persistent strains formed a biofilm slightly better at both 0.5 BHI and 1.5 BHI, but the observed differences were not statistically significant (Figure 5).

Collectively, strains from both groups formed the weakest biofilm at 4 °C, pH 7, 0% NaCl, and 1 BHI, and the strongest at 37 °C, pH 7, 0% NaCl, and 0.5 BHI (Figure 5).

## 4. Discussion

There is a growing demand among consumers for fresh and low-processed foods. The contamination of the fish processing environment with *L. monocytogenes* increases the epidemiological risk linked to fish product consumption. The ability of *L. monocytogenes* to survive in extreme conditions and to form biofilms on various surfaces is a significant challenge for food safety [9]. One of the factors affecting the distribution of pathogens in the facility is the type and quality of the equipment used for material processing. In our study, among the 62 *L. monocytogenes* isolates (8.6%) obtained from processing devices used for fish fillet production, the highest number (14, 22.6%) originated from the filleting machine knives, which directionally contacts with the fish flesh and thus being a probable source of bacteria. A study by Kurpas et al. [8] confirmed food of animal origin as a source of pathogenic *L. monocytogenes* for humans. The presence of persistent strains within the food processing environment entails the contamination of finished products, increasing the risk for future consumers, especially when the product is raw and ready-to-eat fish. Part of the devices included in the processing line have a continuous contact with processed fish. The pin bone remover or knives in the filleting machines can directly contaminate the food, frequently consumed raw or cold-smoked. Salmonidae fish are a popular species consumed without any heat treatment. That is why *L. monocytogenes* infections are enormously dangerous for consumers and should be monitored to prevent outbreaks of listeriosis. Similarly, the studies of Di Ciccio et al. [37] confirmed an overall *L. monocytogenes* prevalence rate of 16% in a cold-smoked salmon processing environment. The samples from working tables (43%) and slicing machines (37%) were the most contaminated [37]. Leong et al. [38] reported a lower *L. monocytogenes* prevalence in the seafood industry compared to the dairy, meat, and vegetable industries. The installation of new equipment increased *L. monocytogenes* occurrence in food production facilities (from 5 to 23% during a year) [38].

The fish processing environment, due to the amount of equipment used during the processing of the raw materials, increases the risk of the finished products being contaminated with *L. monocytogenes*. Despite the procedures used, cleaning and disinfection are becoming insufficient and not effective if we consider the fact of the frequent presence of *L. monocytogenes* persistent strains along the food processing lines. This is one of the most problematic properties of this bacteria, which easily adapts to a wide spectrum of unfavorable factors. They easily adapt to stress factors, form biofilms, and are resistant to disinfectants. In our study, we found six persistent strains of *L. monocytogenes*. Miettinen and Wirtanen [39], analyzing 81 isolates from 15 fish farms and fish processing plants, found 30 *L. monocytogenes* pulsotypes. Scientists observed the repetitive isolation of at least one pulsotype from the same facility, suggesting the presence of persistent *L. monocytogenes* strains in the processing environment. In Ramires et al. [40] research, two of the total four *L. monocytogenes* pulsotypes from salmon sushi were persistent. Aalto-Araneda et al. [41] sampled the same *L. monocytogenes* pulsotypes on separate sampling occasions in three of seven fish processing plants. Three of eight RAPD types of *L. monocytogenes*, found in raw fish and their products from Polish fish processing plants, were collected continually over 8–10 months [42]. Cruz and Fletcher [43] identified persistent *L. monocytogenes* strains in the mussel processing environment (in raw mussels and finished products). One of the persistent pulsotypes was linked to non-perinatal listeriosis cases.

The mechanism of bacterial persistence is poorly understood. Some authors suggest that biofilm-forming ability is an essential factor for its prolonged survival in the food production environment [40]. Our research showed a higher rate of biofilm formation initiation for persistent *L. monocytogenes* strains. Their number, recovered from the biofilm, was statistically significantly higher than for sporadic strains after 1, 2, 3, and 4 h, but not after 5 h of the process. Lundén et al. [26] reported that the persistent *L. monocytogenes* strains revealed higher adherence than the nonpersistent strains after 1 and 2 h of contact time. However, after 72 h, the adherence ability was comparable for persistent and nonpersistent strains [26]. Contrary to Lundén et al., Costa et al. [19] observed the significantly higher attachment abilities of nonpersistent *L. monocytogenes* isolates. In our study, the number of *L. monocytogenes* recovered from biofilms, formed in the control conditions (37 °C, pH 7, 0% NaCl, 1 BHI), for persistent strains was statistically significantly higher (6.85 log CFU × cm^−2^) compared with sporadic strains (6.09 log CFU × cm^−2^). The biofilm formation was the most intense at 37 °C, pH 9, 5% NaCl salinity, and a reduced nutrient availability (0.5 BHI), which confirms our previous studies [44]. Regardless of the thermal conditions and environmental pH, the number of bacteria recovered from the biofilm was statistically significantly higher for the persistent strains. We did not observe statistically significant differences between biofilms formed in environments with various levels of nutrient availability and at 5% NaCl salinity. The tolerance of *L. monocytogenes* to environmental stress factors contributes to a better ability to survive in food products and processing environments. Researchers have suggested that the adaptation of *L. monocytogenes* to stress factors is one of the main theories of the formation of persistent strains. Jensen et al. [45] have reported the enhanced adhesion of *L. monocytogenes* strains to a plastic surface at 37 °C after NaCl addition to the growth medium. Taylor and Stasiewicz [46] documented the growth of persistent and sporadic strains of planktonic *L. monocytogenes* cells at 5% and 10% salt concentration, as well as acidic (pH 5.2) and alkaline (pH 9.2) conditions. Nowak et al. [47] reported increased biofilm formation in such conditions. In turn, Assisi et al. [48] found that the persistence of *L. monocytogenes* in the environment is probably a matter of the poor sanitation of the facility and not the ability of isolates to form a biofilm and tolerance to disinfectants.

Microbial tolerance towards sanitizing agents may lead to the higher persistence of pathogens in the production environment [20]. The results of our study show that the applied disinfectants were effective against persistent and sporadic *L. monocytogenes* strains in concentrations lower than those recommended by the manufacturer. Their high efficacy could be because pathogen cells were in planktonic form. Moreover, no potentially protecting organic substances were present in the environment. Magalhães et al. [23] noted the reduction in persistent and nonpersistent *L. monocytogenes* isolates by commonly used disinfectants applied in concentrations lower than those recommended by the manufacturers. They found no relation between pathogen persistence and increased resistance to sanitizers. Costa et al. [19] also noticed no significant differences between persistent and nonpersistent *L. monocytogenes* isolates in their sensitivity to disinfectant treatments, suggesting no link between persistence and disinfectant susceptibility. In our study, we observed significant differences in susceptibility to Sansept 0200 (Didecyldimethylammonium chloride, benzyl-C12-16-alkyldimethyl chlorides), Peroxat (Peracetic acid, hydrogen peroxide), and Calcium hypochlorite between persistent and sporadic *L. monocytogenes* strains. According to the calculated MBC values, Sansept was the most effective disinfectant and Peroxat the least effective. However, the obtained results do not support that resistance to disinfectants is one of the hypotheses for the formation of persistent strains. Wang et al. [49] found no significant difference in disinfectant tolerance between the persistent and transient strains.

The study also assessed the belonging to serogroups and antimicrobial resistance. The highest number of collected *L. monocytogenes* isolates (28, 73.7%) was classified as 1/2a-3a serogroup. Additionally, Gambarin et al. [50] have shown a high percentage of strains related to serotype 1/2a (73.33%) in RTE seafood. In turn, Ramires et al. [40] have noted that all *L. monocytogenes* isolates from sushi establishments belonged to serotype 4b. Serotype 4b-4d-4e was the second numerous group (15.8% of *L. monocytogenes* isolates) in the present study and included the persistent LMO-P6 strain. The other persistent strains belonged to the 1/2a-3a serogroup. In our study, 18 (47.4%) isolates of *L. monocytogenes* strains were susceptible to all antibiotics tested. The highest number of strains (34.2%) was resistant to meropenem. Three of the six persistent strains were resistant to all tested antibiotics. Skowron et al. [51] have found the highest resistance to erythromycin (47.1%) and cotrimoxazole (47.1%) among *L. monocytogenes* strains isolated from the fish and fish processing plant.

The ability of *L. monocytogenes* to adhere, invade, and grow in intestinal cells is directly associated with the pathogen’s virulence [33,52,53]. The invasiveness of *L. monocytogenes* isolates against HT-29 cells amounted to 7.99% for sporadic strain LMO 61 and 11.21% for persistent strain LMO-P4. We did not observe statistically significant differences between the mean invasiveness values for the persistent and sporadic strains. Moroni et al. [54] have reported an invasion ability of *L. monocytogenes* LSD348 against HT-29 cells of 45.49%. Jensen et al. [45] have observed low invasiveness of the four RAPD type 9 persistent strains (N53-1, H13-1, La111, and M103-1) against Caco-2 cells compared to the remaining strains. Wałecka-Zacharska et al. [33] have noted that *L. monocytogenes* strains of 1/2a serotype revealed a lower ability to invade epithelial cells than those of the 4b and 1/2b serotypes.

There are many controversies about the persistence mechanism of strains frequently and repetitively isolated from the production environment. Research results concerning the relationship between resistance to disinfectants, adherence ability, biofilm formation, and the long-term survival of *Listeria* spp. in food industry plants are contradictory [3,23,55]. According to the hypotheses of Carpentier and Cerf [1], the key factor of a strain’s persistence is the specificity of harborage sites inhabited by the bacteria. The bacteria living in these areas, protected from environmental stresses, can survive for a longer time [19,46].

## 5. Conclusions

The presence of persistent strains of *L. monocytogenes* increases the risk of food cross-contamination. Our study aimed to characterize strains collected from fish processing plants and the different phenotypic responses of persistent and sporadic strains. The results indicate that the persistent strains of *L. monocytogenes* can form a stronger biofilm (also in unfavorable environmental conditions) and have a lower disinfectant susceptibility than sporadic strains. We think that future research should explore the genetic variation between persistent and sporadic strains to explain the molecular basis of persistence.

## Figures and Tables

**Table 1:** Characteristics of the disinfectants used for the evaluation of the minimum bactericidal concentrations.

Group of Disinfectants	Trade Name	Active Substances	Manufacturer	Working Solution Concentration
Quaternary ammonium compounds	Sansept 0200	Didecyldimethylammonium chloride, benzyl-C12-16-alkyldimethyl chlorides	Sanechem	3 mL/L
Oxidizing agents	Peroxat	Peracetic acid, hydrogen peroxide	Agro-trade	5 mL/L
Chlorine compounds	Calcium hypochlorite	Hypochlorous acid calcium salt	Chem Point	2 g/L
Iodine compounds	Rapicid	Iodine	Pfizer	10 mL/L

**Table 2:** Experimental conditions regarding the set of temperature, pH, salinity, and nutrient availability in individual variants of biofilm formation.

Environment Parameter	Experimental Conditions Set for Biofilm Formation	Temperature (°C)	pH	Salinity (% NaCl)	Nutrient Availability (BHI)
Temperature (°C)	1	4	7	0	1.0
	2	20	7	0	1.0
	**3**	**37**	**7**	**0**	**1.0**
pH	4	37	4	0	1.0
	**5**	**37**	**7**	**0**	**1.0**
	6	37	9	0	1.0
Salinity (% NaCl)	**7**	**37**	**7**	**0**	**1.0**
	8	37	7	5	1.0
	9	37	7	10	1.0
Nutrient availability (BHI)	10	37	7	0	0.5 *
	**11**	**37**	**7**	**0**	1.0 *
	12	37	7	0	1.5 *

BHI—brain heart infusion broth; * BHI 1.0—medium containing the amount recommended by the manufacturer; BHI 0.5—medium containing 50% of the amount recommended by the manufacturer; BHI 1.5—medium containing 150% of the amount recommended by the manufacturer. The control variant was marked with bold and the variable parameters with grey color.

**Table 3:** Sources of *L. monocytogenes* isolates collected during the research.

Element of the Processing Line		Number (%) of All Isolates	Isolates *
Box pallets in the raw material warehouse		3 (4.8)	LMO 36, LMO 39, LMO 62
Head remover machine (cutting element)		7 (11.3)	**LMO-P2** (LMO 5, LMO 14, LMO 20, LMO 45) LMO 48, LMO 55, LMO 61
Filleting machine	Conveyor	3 (4.8)	LMO 40, LMO 49, LMO 52
	Knives	14 (22.6)	**LMO-P3** (LMO 7, LMO 16, LMO 33, LMO 41, LMO 54), **LMO-P4** (LMO 9, LMO 18, LMO 26, LMO 43, LMO 60) LMO 2, LMO 13, LMO 51, LMO 53
Trim conveyor	Conveyor rollers	7 (11.3)	**LMO-P1** (LMO 3, LMO 24, LMO 35, LMO 56) LMO 17, LMO 25, LMO 59
	Worktops	3 (4.8)	LMO 23, LMO 31, LMO 46
Pin bone remover	Conveyor	3 (4.8)	LMO 6, LMO 21, LMO 42
	Trommel	4 (6.5)	**LMO-P6** (LMO 22, LMO 38, LMO 44) LMO 58
Skin remover machine		8 (12.9)	**LMO-P5** (LMO 15, LMO 27, LMO 47, LMO 50) LMO 8, LMO 32, LMO 34, LMO 57
Fillet washer		3 (4.8)	LMO 11, LMO 29, LMO 30
Portion machine	Conveyor	4 (6.5)	LMO 1, LMO 10, LMO 19, LMO 28
	Knives	3 (4.8)	LMO4, LMO 12, LMO 37

* In brackets are isolates clustered as belonging to each persistent strain written in bold letters.

**Table 4:** Antibiotic resistance profiles.

Profile Number	Antibiotic Resistance Profile	Number (%) of Strains	Isolates and Strains Representing the Profile
1	R: --- S: P, AM, MEM, E, SXT	18 (47.4) ^a^	LMO 2, LMO 6, LMO 8, LMO 10, LMO 11, LMO 19, LMO 21, LMO 25, LMO 28, LMO 29, LMO 32, LMO 36, LMO 37, LMO 40, LMO 42, LMO 51, LMO 57, LMO 62
2	R: AM S: P, MEM, E, SXT	5 (13.2) ^b^	LMO 1, LMO 13, LMO 17, LMO 49, LMO 55
3	R: MEM, E, SXT S: P, AM	5 (13.2) ^b^	LMO-P1 *, LMO-P2 LMO 23, LMO 46, LMO 61
4	R: P, MEM S: AM, E, SXT	4 (10.5) ^b^	**LMO-P6** LMO 4, LMO 52, LMO 58
5	R: P, AM, MEM, E, SXT S: ---	4 (10.5) ^b^	**LMO-P3, LMO-P4, LMO-P5** LMO 53
6	R: SXT S: P, AM, MEM, E	2 (5.3) ^b^	LMO 48, LMO 59

* bold—persistent strains; P—penicillin; AM—ampicillin; MEM—meropenem; E—erythromycin; SXT—cotrimoxazole; R—resistant; S—sensitive; ^a^,^b^—statistical significance with *p* ≤ 0.05.

**Table 5:** Invasiveness of the persistent and sporadic strains of *L. monocytogenes* against HT-29 cells.

Persistent Strains		Sporadic Strains	
Strain	%	Strain	%
LMO-P1	3.42 ± 0.70 ^a,b^	LMO 4	1.42 ± 0.17 ^a^
LMO-P2	2.05 ± 0.35 ^a^	LMO 23	5.59 ± 1.14 ^b^
LMO-P3	9.55 ± 4.37 ^c^	LMO 46	4.25 ± 0.93 ^b^
LMO-P4	11.21 ± 3.19 ^d^	LMO 52	2.10 ± 0.69 ^a^
LMO-P5	5.71 ± 1.66 ^b^	LMO 53	6.22 ± 1.84 ^b,e^
LMO-P6	1.07 ± 0.39 ^a^	LMO 61	7.99 ± 1.11 ^c,e^
Mean	5.50 ± 3.71 *	Mean	4.60 ± 2.29 *

^a^,^b^,^c^,^d^,^e^, values marked with different letters differ statistically (*p* ≤ 0.05). * values marked with this symbol do not differ statistically (*p* ≤ 0.05).

**Table 6:** Minimum bactericidal concentration value of the tested disinfectants against the persistent and sporadic strains of *L. monocytogenes*.

Disinfectant	PERSISTENT STRAINS							SPORADIC STRAINS						
	LMO-P1	LMO-P2	LMO-P3	LMO-P4	LOM-P5	LMO-P6	MEAN	LMO 4	LMO 23	LMO 46	LMO 52	LMO 53	LMO 61	MEAN
Sansept 0200 [mL/L]	0.81 ± 0.11 (27%)	0.81 ± 0.18 (27%)	1.17 ± 0.27 (39%)	1.47 ± 0.31 (49%)	0.93 ± 0.09 (31%)	0.72 ± 0.15 (24%)	0.99 ± 0.28 ^a^ (33%)	0.15 ± 0.04 (5%)	0.21 ± 0.10 (7%)	0.21 ± 0.06 (7%)	0.18 ± 0.07 (6%)	0.27 ± 0.02 (9%)	0.30 ± 0.12 (10%)	0.22 ± 0.06 ^b^ (7%)
Peroxat [mL/L]	3.50 ± 1.02 (70%)	3.10 ± 0.68 (62%)	4.10 ± 0.91 (82%)	4.60 ± 1.16 (92%)	3.85 ± 0.74 (77%)	3.10 ± 1.00 (62%)	3.71 ± 0.59 ^a^ (74%)	1.75 ± 0.51 (35%)	2.10 ± 0.43 (42%)	2.05 ± 1.40 (41%)	1.85 ± 0.82 (37%)	2.50 ± 0.99 (50%)	2.90 ± 0.38 (58%)	2.19 ± 0.43 ^b^ (44%)
Calcium hypochlorite [g/L]	0.68 ± 0.23 (34%)	0.54 ± 0.10 (27%)	0.78 ± 0.31 (39%)	0.90 ± 0.09 (45%)	0.80 ± 0.66 (40%)	0.60 ± 0.17 (30%)	0.72 ± 0.14 ^a^ (36%)	0.30 ± 0.08 (15%)	0.34 ± 0.13 (17%)	0.38 ± 0.20 (19%)	0.34 ± 0.14 (17%)	0.48 ± 0.07 (24%)	0.40 ± 0.23 (20%)	0.37 ± 0.06 ^b^ (19%)
Rapicid [mL/L]	3.10 ± 0.71 (31%)	3.20 ± 0.52 (32%)	3.80 ± 0.84 (38%)	4.00 ± 1.11 (40%)	3.80 ± 0.95 (38%)	2.70 ± 0.36 (27%)	3.43 ± 0.51 ^a^ (34%)	2.80 ± 0.29 (28%)	3.10 ± 0.41 (31%)	3.10 ± 0.37 (31%)	3.00 ± 0.19 (30%)	3.10 ± 0.28 (31%)	3.60 ± 0.37 (36%)	3.12 ± 0.26 ^a^ (31%)

A percentage of working solution (given in brackets) concentration, which is the MBC value; ^a^,^b^, values marked with different letters differ statistically with *p* ≤ 0.05.

## Data Availability

The datasets generated for this study are available on request to the corresponding author.
